# Health-related quality of life of medical students in a Brazilian student loan programme

**DOI:** 10.1007/s40037-016-0283-3

**Published:** 2016-07-20

**Authors:** Liliane Lins, Fernando Martins Carvalho, Marta Silva Menezes, Larissa Porto-Silva, Hannah Damasceno

**Affiliations:** 1 Bahiana School of Medicine and Public Health, Salvador, Bahia Brasil; 2 School of Medicine, Federal University of Bahia, Salvador, Bahia Brasil

**Keywords:** Quality of life, Medical students, Government financing, Medical education

## Abstract

This study aimed to evaluate the health-related quality of life of medical students participating in a large Brazilian government loan programme for undergraduate students in private schools.

A cross-sectional study in a stratified sample of students from a private medical school in Salvador, Brazil, evaluated their health-related quality of life by using a Brazilian Portuguese version of the 36-item Short Form Health Survey questionnaire (SF-36).

Students supported by the loan programme consistently presented lower mean scores in all SF-36 domains and in the physical and mental component summary scores than those who were not in the programme. Students supported by the loan programme presented systematically lower physical and mental component mean scores, after stratification by age, gender, school year, physical activity, sleepiness, headache, having a car, having a housemaid, living with family, and living in a rented house.

The loan programme has enabled less wealthy undergraduate students to attend private medical schools in Brazil. However, this support is insufficient to improve students’ health-related quality of life during medical school, as compared with students who do not participate in the programme. Because of a poorer health-related quality of life, students supported by the loan programme deserve special attention from private medical schools.

## What this paper adds

- In 2001, the Brazilian government launched a loan programme for undergraduate students in private institutions. Each year, approximately 22,000 medical students participate in the programme. Little or nothing is known about the impact of such loan programmes upon the health-related quality of life of medical students. The programme has enabled less wealthy students to attend private medical schools. However, during the six years of medical school, students in the loan programme were unable to improve their health-related quality of life as compared with that of students who did not participate in the programme.

## Introduction

All over the world, future undergraduate medical students undertake intensive preparation to succeed in the highly competitive selection process. While being enrolled in a six-year undergraduate course in medicine, students encounter many sources of stress, including academic pressure, sleep deprivation, work overload, financial concerns, exposure to patient death and suffering, a competitive work environment [1], pressure to learn a large quantity of new information, time pressure, and loss of opportunities for participation in social activities [2]. It has been documented that students’ mental health may deteriorate during medical school [3]. It is reasonable to hypothesize that medical students’ health-related quality of life can be impaired during school, particularly among individuals with a lower socioeconomic status. Medical schools should provide institutional programmes for evaluating and improving students’ well-being and health-related quality of life.

In 2013, there were 111,198 medical students in Brazil [4]. In 2014, there were 240 medical schools in the country, 107 (44.6 %) public and 133 (55.4 %) private, offering 8,644 and 12,059 openings for new students, respectively [5].

In 2012, approximately 7,000 of the 17,000 new medical students in Brazil were supported by a federal government loan programme for undergraduate students in private institutions (FIES – ‘*Fundo de Financiamento ao Estudante do Ensino Superior*’). Each year, approximately 22,000 medical students participate in the programme [6].

The Brazilian Ministry of Education created the student loan programme in 2001. Students with a positive score on a nationwide evaluation of postsecondary education, conducted by the Ministry of Education, may apply for a loan. Financing varies from 50 % to 100 % of the monthly tuition fees depending on the proportion (20 to 60 %) of the monthly per capita family income associated with this expenditure. Financing instalments are fixed and monthly. The interest rate is 3.4 % per year, with a grace period of 18 months after the end of the financing period. The repayment term is up to three times the duration of study plus 12 months [7].

From 2010 to 2014, the Brazilian government celebrated that fact that more than one million undergraduate students had been supported by the loan programme, having invested approximately 44 billion reais (Brazilian currency) equivalent to 15 billion American dollars [8].

This study is part of broader research that investigated several aspects of the health-related quality of life of students from a private medical school [9]. Specifically, we aimed to evaluate the health-related quality of life of undergraduate medical students participating in a Brazilian government student loan programme compared with that of students not participating in this programme.

## Methods

### Study design and population

A cross-sectional study was conducted in medical students from the Bahiana School of Medicine and Public Health, a private institution in the city of Salvador, State of Bahia, Brazil. The medical course started in 1953; currently, it offers 95 new openings each semester. The entire course takes six years on a full-time basis, corresponding to 7,716 hours, and results in a generalist physician diploma. The total annual tuition is 38,220.00 reais (Brazilian currency) or 13,080.00 American dollars [10].

A total sample size of 180 was arbitrarily and conveniently defined, according to human and financial resources. A stratified sampling procedure was used to randomly select 30 students from each of the six classes. While inside the medical school premises, each student was invited to complete a self-administered questionnaire comprising the SF-36 form and information on sociodemographic data, housing, logistics and financing structure, family support, and other aspects of his/her health-related quality of life (e. g., sleepiness, defined as a score of 10 or more points on the Epworth Sleepiness Scale [11], headache, and regular physical activity).

Data were collected from October to November 2013 during or after classes. The research team that supervised the data collection asked the students repeatedly to complete all fields of the questionnaire. If a student returned a questionnaire with a blank field, he/she was immediately asked to kindly provide the missing information.

### Data measurement

The 36-item Short Form Health Survey questionnaire (SF-36) is frequently used to evaluate health-related quality of life. On 11 April 2016, a PubMed search using the term ‘SF-36 survey’ yielded 10,603 items.

The Brazilian Portuguese [12] version of the SF-36 Health Survey version 1, as recommended by QualityMetric Incorporated [13], was used in this study. The 4‑week recall form has 36 items, but one self-evaluated transition item is not used in the construction of its eight multi-item scales: physical functioning, role limitations due to physical problems, bodily pain, general health perceptions, vitality, social functioning, role limitations due to emotional problems and mental health. The eight multi-item scales were aggregated into a physical component (PCS) and a mental component summary (MCS) score. All eight of the SF-36 scales contribute, with different weights, to the PCS and MCS scores. However, physical functioning, role limitations due to physical problems, and bodily pain contribute more significantly to the PCS, whereas social functioning, role limitations due to emotional problems, and mental health contribute more significantly to MCS. The vitality, general health perceptions, and social functioning scales contribute to both the PCS and MCS scores.

The scoring of the eight scales using the original 0 to 100 algorithms (raw scores) and the two summary components was performed by QualityMetric Health Outcomes^TM^ Scoring Software 4.0 based on norms with a mean of 50 and a standard deviation of 10 [13]. Normalized scores below 50 should be interpreted as below the US general population T‑score, and scores above 50 can be interpreted as above the US general population T‑score. Meaningful comparisons can be directly made across the scale and component scores because they have the same variance. Therefore, normalized scores allow unbiased identification and quantification of the SF-36 domains that are most affected by specific risk factors. Higher scores represent a better health-related quality of life. This study was licensed by QualityMetric Health Outcomes^TM^ with the number QM025904.

### Statistical analysis

The Mann-Whitney test was used to compare the distributions of the eight SF-36 scales and the two composite scores (PCS and MCS), stratified according to student loan programme support and the school year.

Simple linear regression analysis yielded regression coefficients for the relationship between the mean PCS and MCS scores and the school year according to student loan programme strata.

T-tests for independent samples were used to compare the mean PCS and MCS scores, stratified according to student characteristics and loan programme support. The same test was used to compare the eight mean SF-36 scores according to living arrangements: living in a rented house, living with the family, having a housemaid, and having a car.

Two-way analysis of variance was used to compare the means of the SF-36 scales and their component scores according to gender and student loan programme support, to identify the main effects of each factor, and to evaluate the interactions among them.

Data were analyzed using the Statistical Package for the Social Sciences (SPSS) version 21.0.

### Ethical aspects

The study was approved by the Ethics Review Board of Bahiana School of Medicine and Public Health (Registration Number 233794/2013) in accordance with the Brazilian Health National Council Resolution 466/12. All students were informed and signed the two copies of the consent form approved by the Ethics Board. A copy of the form stayed with the student.

## Results

A total of 182 students returned their questionnaires. There were no formal refusals to fill out the questionnaires. Two questionnaires were discarded because of incomplete and incongruent data. Out of the 180 students included in the final sample, 93 were supported by the loan programme and 87 were not.

The normalized scores of the eight domains of the SF-36 and the component scores for the 180 students revealed that the physical functioning score, bodily pain score, and PCS were slightly above the mean normal scores (50.0). The scores for role limitations due to emotional problems and social functioning, and the MCS were particularly low (41.6 ± 13.6; 42.7 ± 10.7, and 42.0 ± 11.7), respectively. The 93 students supported by the loan programme consistently presented with lower scores in all SF-36 domains and component summaries than the 87 students who were not in the programme (Table 1).

**Table 1:** SF-36 health-related quality of life raw and normalized scores (mean ± SD) according to participation in a student loan programme in 180 students from a private medical school in Salvador, Brazil, in 2013

Domain/Component score	Normalized score				Raw score
	Loan programme		*P*	Total (*n* = 180)	Total (*n* = 180)
	No (*n* = 87)	Yes (*n* = 93)			
Physical functioning	53.6 ± 5.8	49.5 ± 7.2	0.001	51.5 ± 6.8	86.5 ± 16.3
Role physical	47.1 ± 10.5	42.9 ± 11.6	0.010	44.9 ± 11.3	60.0 ± 39.8
Bodily pain	50.6 ± 9.1	49.7 ± 9.1	0.508	50.2 ± 9.1	70.6 ± 21.2
General health	48.3 ± 6.3	44.2 ± 7.5	0.001	46.2 ± 7.2	61.9 ± 15.4
Vitality	48.1 ± 9.5	45.4 ± 9.3	0.053	46.7 ± 9.5	50.0 ± 20.1
Social functioning	44.2 ± 10.0	41.4 ± 11.1	0.073	42.7 ± 10.7	66.9 ± 24.5
Role emotional	45.1 ± 12.8	38.4 ± 13.7	0.001	41.6 ± 13.6	56.5 ± 43.2
Mental health	47.1 ± 9.4	44.8 ± 10.2	0.111	45.9 ± 9.9	67.9 ± 17.4
PCS	51.8 ± 6.6	49.2 ± 7.0	0.010	50.4 ± 6.9	–
MCS	43.8 ± 11.7	40.2 ± 11.4	0.037	42.0 ± 11.7	–

*PCS* Physical Component Score, *MCS* Mental Component Score

The mean PCS of students supported by the loan programme, compared with that of the students not supported by the programme, was slightly lower in the first year of the medical course. Students in the loan programme presented much lower PCS scores in the third and sixth years compared with those who did not participate in the programme. The MCS scores of the students participating in the loan programme were consistently lower than those who did not participate, in all six years of the medical course (Table 2).

**Table 2:** SF-36 PCS and MCS normalized scores (mean ± SD) according to participation in a student loan programme support and school year of students from a private medical school in Salvador, Brazil, in 2013

Component	Loan programme	1^st^ Year (*n* = 28)	2^nd^ Year (*n* = 31)	3^rd^ Year (*n* = 32)	4^th^ Year (*n* = 32)	5^th^ Year (*n* = 26)	6^th^ Year (*n* = 31)
PCS	No	49.9 ± 5.9	50.3 ± 6.5	53.6 ± 5.0	49.2 ± 8.1	51.7 ± 5.8	55.4 ± 6.0
	Yes	49.8 ± 7.1	52.5 ± 7.8	45.6 ± 7.8	50.1 ± 4.8	50.9 ± 4.9	47.5 ± 6.7
MCS	No	48.4 ± 5.3	41.8 ± 12.9	41.1 ± 12.0	39.3 ± 13.4	51.9 ± 6.3	44.4 ± 11.2
	Yes	43.2 ± 10.3	37.8 ± 9.5	40.6 ± 12.5	38.0 ± 10.7	45.5 ± 12.3	35.5 ± 12.0

*PCS* Physical Component Score, *MCS* Mental Component Score

Straight lines fitted to mean PCS or MCS scores, according to the school year of the students not participating in the loan programme showed weak positive slopes (0.834 ± 0.424 and 0.345 ± 0.763, respectively). However, the lines fitted to the mean PCS or MCS scores showed weak negative slopes (−0.171 ± 0.422 and −0.543 ± 0.694, respectively) among students who did participate in the programme.

In the first and sixth years of the medical course, students in the loan programme, compared with those not in the programme, consistently presented lower scores for the eight domains of the SF-36, and the PCS and MCS scores. The only exception was the mean score for role limitations due to physical problems, which was slightly higher among students in the first year supported by the loan programme. Associations between mean SF-36 scores and loan programme support were stronger in the sixth year than in the first year, as revealed by higher significant *P*-values for these comparisons in the last year of the medical course. In the sixth year of school, students in the loan programme presented particularly low mean scores for role limitations due to physical problems (35.5 ± 8.1), social functioning (37.4 ± 10.3), role limitations due to emotional problems (34.3 ± 12.4), and MCS (35.5 ± 12.0) (Table 3).

**Table 3:** SF-36 health-related quality of life normalised scores (mean ± SD) according to participation in a student loan programme among students from the first and the sixth year of course from a private medical school in Salvador, Brazil in 2013

Domain/Component score	Loan programme	1st Year (*n* = 28)^a^	*P* ^c^	6th Year (*n* = 31)^b^	*P* ^d^
Physical functioning	No	53.8 ± 3.4	0.024	55.9 ± 2.1	0.012
	Yes	48.5 ± 6.2		50.7 ± 6.1	
Role physical	No	42.1 ± 10.0	0.464	50.8 ± 7.7	0.001
	Yes	45.2 ± 10.1		35.5 ± 8.1	
Bodily pain	No	51.3 ± 11.2	0.944	52.8 ± 7.7	0.077
	Yes	52.3 ± 8.3		46.3 ± 9.3	
General health	No	50.2 ± 4.6	0.027	50.0 ± 8.1	0.021
	Yes	43.9 ± 7.2		43.5 ± 6.2	
Vitality	No	49.1 ± 6.2	0.308	52.4 ± 11.1	0.036
	Yes	45.9 ± 7.6		43.3 ± 10.7	
Social functioning	No	49.5 ± 6.9	0.089	47.4 ± 8.6	0.010
	Yes	41.5 ± 11.9		37.4 ± 10.3	
Role emotional	No	49.0 ± 10.2	0.229	43.0 ± 13.6	0.109
	Yes	42.5 ± 12.8		34.3 ± 12.4	
Mental health	No	48.4 ± 5.0	0.832	48.7 ± 8.1	0.015
	Yes	47.8 ± 7.7		40.2 ± 10.8	
PCS	No	49.9 ± 5.9	0.494	55.4 ± 5.9	0.002
	Yes	48.8 ± 7.1		47.5 ± 6.7	
MCS	No	48.4 ± 5.3	0.226	44.4 ± 11.2	0.029
	Yes	43.2 ± 10.3		35.5 ± 12.0	

*PCS* Physical Component Score, *MCS* Mental Component Score

^a^FIES No = 10; Yes = 18

^b^FIES No = 17; Yes = 14

^c^Mann-Whitney test: FIES No vs. FIES Yes in the 1st year stratum

^d^Mann-Whitney test: FIES No vs. FIES Yes in the 6th year stratum

PCS and MCS scores were consistently lower among students in the loan programme compared with students not in the programme regardless of the following factors: age, gender, sleepiness, headache, regular physical activity, living in a rented house, living with family, having a housemaid, and having a car (Table 4).

**Table 4:** SF-36 health-related quality of life normalised component scores (mean ± SD) according to covariates and participation in a student loan programme in 180 students from a private medical school in Salvador, Brazil, in 2013

Covariates		*N*	PCS		*P*	MCS		*P*
			Loan programme			Loan programme		
			No	Yes		No	Yes	
Age, years	17–22	93	50.7 ± 6.8	48.8 ± 7.3	0.202	43.4 ± 11.5	40.9 ± 10.7	0.279
	23–33	87	52.9 ± 6.3	49.5 ± 6.5	0.015	44.3 ± 11.9	39.4 ± 12.3	0.066
Gender	F	105	50.9 ± 6.9	48.1 ± 7.3	0.048	42.7 ± 11.5	38.6 ± 11.5	0.075
	M	75	53.4 ± 5.8	50.5 ± 6.3	0.045	45.7 ± 11.8	42.1 ± 11.2	0.181
Sleepiness	No	87	53.7 ± 6.0	50.1 ± 6.7	0.009	44.1 ± 11.9	42.1 ± 11.9	0.009
	Yes	93	50.0 ± 6.8	48.3 ± 7.2	0.267	43.5 ± 11.5	38.5 ± 10.9	0.031
Headache	No	84	53.2 ± 5.9	51.1 ± 6.2	0.126	45.0 ± 11.2	43.0 ± 10.1	0.397
	Yes	96	50.3 ± 7.1	47.8 ± 7.2	0.095	42.5 ± 12.1	38.3 ± 12.0	0.092
Physical activity	No	73	50.6 ± 7.3	48.9 ± 7.0	0.350	41.8 ± 13.6	38.0 ± 11.0	0.193
	Yes	107	52.4 ± 6.2	49.3 ± 6.9	0.019	44.8 ± 10.6	42.3 ± 11.5	0.243
Rented house	No	142	51.9 ± 6.4	48.9 ± 7.6	0.011	43.5 ± 12.1	41.3 ± 11.5	0.268
	Yes	38	50.9 ± 8.4	49.7 ± 5.4	0.694	46.4 ± 6.8	37.7 ± 11.1	0.026
Lives with family	No	48	52.7 ± 6.1	49.5 ± 5.3	0.062	41.9 ± 10.5	39.3 ± 12.0	0.441
	Yes	132	51.5 ± 6.8	49.0 ± 7.6	0.042	44.4 ± 12.0	40.6 ± 11.2	0.066
Has a housemaid	No	74	51.4 ± 6.0	49.1 ± 6.6	0.168	43.4 ± 10.9	40.2 ± 11.9	0.291
	Yes	106	52.0 ± 6.9	49.2 ± 7.4	0.052	44.0 ± 12.0	40.2 ± 11.0	0.102
Has a car	No	59	54.2 ± 6.5	49.2 ± 7.4	0.012	42.3 ± 13.1	38.6 ± 10.2	0.235
	Yes	121	51.0 ± 6.5	49.1 ± 6.7	0.119	44.3 ± 11.2	41.2 ± 12.1	0.146

*PCS* Physical Component Score, *MCS* Mental Component Score

Among the individuals without sleepiness, the mean PCS and MCS scores were significantly lower (*p* = 0.009, in both situations) among the students participating in the loan programme compared with students who did not participate (Table 4).

Having a housemaid was more frequent among the 87 students not participating in the loan programme (73.6 %) than among the 93 students who participated in the programme (45.2 %). However, having or not having a housemaid was not significantly associated with the PCS (49.2 ± 7.4 vs. 49.1 ± 6.7) or MCS (40.2 ± 11.9 vs. 40.2 ± 11.0) score among the students supported by the loan programme. Similar tendencies were observed in the scores of the students who were not supported by the programme (Table 4).

Of the students, 67 % (121/180) had a car. Having a car was more frequent among the 87 students not participating in the loan programme (74.7 %) than among the 93 students who participated in the programme (60.2 %). Among the 59 students who did not have a car, the mean PCS was significantly higher (*p* = 0.012) among the students not participating in the loan programme (54.2 ± 6.5) than among those who participated (49.2 ± 7.4).

Female students supported by the loan programme presented the lowest mean scores for all SF-36 scales while male students not supported by the programme presented the highest ones. Male students supported by the loan programme and female students not supported by the programme presented intermediate and similar mean scores. Two-way analysis of variance revealed that both gender and participation in the loan programme had independent and significant (*p* < 0.05) main effects on SF-36 scores, except for scores for social functioning and role limitations due to emotional problems. No interaction (*p* = 0.478 was the lowest *p*-value) was found between gender and participation in the loan programme with respect to the means of the ten SF-36 scales (Table 5).

**Table 5:** SF-36 health-related quality of life normalized scores (mean ± SD) according to participation in a student loan programme and gender of 180 students from a private medical school in Salvador, Brazil in 2013

Domain/Component	Loan + female (*n* = 51)	Loan + male (*n* = 42)	Non-loan + female (*n* = 54)	Non-Loan + male (*n* = 33)	*P*_Sex_	*P*_Loan_
Physical functioning	47.9 ± 7.5	51.5 ± 5.8	52.5 ± 6.7	55.4 ± 3.0	0.001	0.001
Role physical	42.0 ± 11.5	44.0 ± 11.7	46.0 ± 10.7	49.0 ± 10.1	0.145	0.008
Bodily pain	47.8 ± 8.9	52.1 ± 9.0	49.4 ± 9.6	52.6 ± 7.9	0.006	0.430
General health	43.5 ± 6.8	45.1 ± 8.2	47.7 ± 5.8	49.2 ± 8.1	0.126	0.001
Vitality	43.7 ± 8.7	47.4 ± 9.8	47.0 ± 8.8	50.0 ± 10.5	0.018	0.041
Social functioning	40.0 ± 11.2	43.1 ± 10.0	44.5 ± 10.6	43.8 ± 8.9	0.458	0.105
Role emotional	35.9 ± 13.4	41.3 ± 13.7	44.4 ± 12.8	46.1 ± 12.8	0.080	0.356
Mental health	43.7 ± 9.8	44.8 ± 10.5	45.6 ± 9.2	49.6 ± 9.5	0.034	0.069
PCS	48.1 ± 7.3	50.5 ± 6.3	50.9 ± 6.9	53.4 ± 5.8	0.017	0.006
MCS	38.6 ± 11.5	42.1 ± 11.2	42.4 ± 11.5	45.7 ± 11.7	0.062	0.030

*PCS* Physical Component Score; *MCS* Mental Component Score

## Discussion

We analyzed our data using normalized scores, following the recommendations of the SF-36 developers, instead of the 0–100 raw scoring system, where 0 is the lowest and 100 is the highest possible score on each scale. This procedure allows to make appropriate, adjusted comparisons between scores from the various SF-36 domains [13].

The students participating in the loan programme, compared with those who did not participate, presented lower normalized mean scores for all SF-36 domains. Indeed, the variable ‘participation in the student loan programme’ can be taken as a proxy of monthly per capita family income. It is reasonable to suppose that a higher family income is associated with, at least to some extent, a better health-related quality of life [14]. However, a study in Brazilian students [15] revealed no correlations between annual family income and PCS (*p* = 0.97) and MCS scores (*p* = 0.19) of the SF-36.

Among 364 Iranian medical students [16], the MCS increased according to family income strata (low, medium, high), but the finding was not statistically significant at the 5 % level. The PCS, however, decreased among individuals in the medium (odds ratio (OR) = 0.25; *p* = 0.034) and high (OR = 0.50; *p* = 0.310) strata of family income. It is important to note that in the Iranian study [11] simple averages of four scales were calculated in order to obtain the PCS (physical functioning, role limitations due to physical problems, bodily pain, and general health perceptions) and MCS (vitality, social functioning, role limitations due to emotional problems, and mental health), scores respectively. However, the correct calculation of the PCS and the MCS requires the use of special algorithms defined by SF-36 developers [17].

Our data revealed that students participating in the loan programme, compared with students not participating in the programme, presented lower mean PCS and, particularly, MCS scores during medical school. Differences in health-related quality of life indicators reached a maximum in the sixth year of school, in which students participating in the programme presented very low scores for role limitations due to physical problems, social functioning, and role limitations due to emotional problems, as well as MCS scores. A longitudinal study with 73 students in the final year of the medical course at a Canadian university revealed a significant deterioration in vitality, role limitations due to physical problems, and role limitations due to emotional problems [18]. Other studies reported that students in the sixth year, compared with those in the first year, had lower PCS [19] and lower PCS and MCS scores [15].

The negative effect of the socioeconomic burden on health-related quality of life is evident among students participating in the loan programme during all the six years of school. However, in the final year of the course, students who were supported by the programme foresee several future responsibilities and challenges, namely: the struggle to obtain a position in the competitive labour market, preparation for the medical residency selection process, and, within a few years, payment of the programme loan. These facts could explain the much lower mental component of their health-related quality of life.

The PCS and MCS scores were consistently lower among students participating in the loan programme, compared with students not participating in the programme, across all strata of several variables.

Female gender, not living with family, living in a rented house, and lack of physical activity are factors known to be strongly associated with poor health-related quality of life. One study [19] in Iranian medical students revealed that lower PCS and MCS scores were associated with female gender, not living with family, and lack of physical activity. Another study [16] in Iranian medical students reported lower MCS scores associated with a lack of regular physical exercise. Male and female students presented similar scores in the eight domains of the SF-36.

Female Brazilian medical students presented lower a MCS than males [15]. Among university students from Serbia (12 % of whom were medical students), PCS and MCS scores were lower in females and in those who did not live with family [20]. In our study, female students supported by the loan programme presented the worst health-related quality of life scores.

The students not participating in the loan programme more frequently had a housemaid or a car than the students who participated in the programme. These are expected findings, as students who do not participate in the loan programme are generally more wealthy and have more access to these types of social and economic support. However, these conditions were not associated with a better health-related quality of life. In the study population, 106 individuals (58.9 %) had a housemaid, a very common situation in Northeast Brazil. Being a housemaid is the most common occupation of working women in Brazil [21], with 6.6 million people employed in this occupation in 2011 [22].

Of the students, 67 % had a car. In young middle-class Brazilians, similar to these medical students, having a car is a symbol of wealth but it is also a useful resource for transportation to the school campus and to cope with curricular demands.

Sleepiness and headache were associated with lower mean PCS and MCS scores, particularly among the students participating in the loan programme. Sleepiness and headache complaints were frequent (51.7 % and 53.3 %, respectively) in our study population, corroborating findings from other studies in Brazilian medical students [23, 24].

## Strengths and weaknesses of this study

Cross-sectional studies have intrinsic limitations. Conclusions based on comparing SF-36 scores of students in the first and sixth years do not have the same strength as those derived from cohort studies.

The small sample size of our study can lead to a type II error. However, our study was able to detect consistent differences in mean SF-36 scores between students enrolled or not enrolled in the loan programme.

This study yielded simple, subjective evaluations of some covariates, such as regular physical activity and headaches. Perhaps the use of the International Physical Activity Questionnaire (IPAQ) and Headache Impact Test (HIT-6), respectively, could improve measurement validity. A strength of this study was the use of the Epworth Sleepiness Scale for evaluating sleepiness.

This exploratory study did not evaluate additional covariates that could influence health-related quality of life in the study population, such as the frequency of organic and mental diseases, the quality of personal relationships with parents and friends, and the occurrence of paramount life events, such as the loss of close relatives and unexpected unemployment.

Students could modify their answers to the questionnaire depending on their awareness of being evaluated (Hawthorne effect). In the context of a cross-sectional study, the Hawthorn effect can only be minimized, but not eliminated. We took precautionary measures to reduce it, by clearly communicating the object of the study to the participants, being discreet in our observations, and by reducing to the minimum number of measurements used in the study.

Finally, caution should be used in generalizing the results of this study to other private medical schools. The Bahiana School of Medicine and Public Health has one of the lowest tuition fees in Brazil, and this factor can act as a selective factor for its student body.

## Conclusions

The student loan programme, run by the Brazilian Government, has enabled less wealthy students to attend private medical schools. However, such support is not sufficient to improve students’ health-related quality of life during medical school compared with that of students who did not participate in the programme.

Students participating in the loan programme presented systematically lower mean health-related quality of life scores, even after stratification by age, gender, school year, physical activity, sleepiness, headache, having a car, having a housemaid, living with family, and living in a rented house. Female students who participate in the loan programme presented particularly low mean scores.

During the six years of medical school, students supported by the loan programme are unable to free themselves of the effects of the low socioeconomic status on their health-related quality of life.

Student loan programmes play an unquestionable role in the educational model in Brazil and in other countries. Our study showed that students supported by the loan programme have poorer health-related quality of life. Therefore, this group deserves special attention from private medical schools.

## References

[1] Dyrbye LN, Thomas MR, Huntington JL (2006). Personal life events and medical student burnout: a multicentre study. Acad Med.

[2] Enns MW, Cox BJ, Sareen J, Freeman P (2001). Adaptive and maladaptive perfectionism in medical students: a longitudinal investigation. Med Educ.

[3] Dyrbye LN, Thomas MR, Shanafelt TD (2006). Systematic review of depression, anxiety, and other indicators of psychological distress among U.S. and Canadian medical students. Acad Med.

[4] INEP (2013). Instituto Nacional de Estudos e Pesquisas Educacionais Anísio Teixeira. Censo da Educação Superior 2013. Brasil.

[5] Antonio Celso Nunes Nassif. Escolas Médicas do Brasil. http://www.escolasmedicas.com.br/estado.php. Accessed 12 July 2015.

[6] Portal Brasil (2013). Médicos e Professores poderão abater dívida do Fies com trabalho na rede pública. Brasil.

[7] Ministério da Educação. FIES. Programa de Financiamento Estudantil. Brasil. http://sisfiesportal.mec.gov.br/. Accessed 13 June 2016.

[8] FIES2015BR. Fies 2015 – Cadastro, Aditamento e inscrições. http://www.fies2014br.com.br. Accessed 12 July 2015.

[9] Lins L, Carvalho FM, Menezes MS, Porto-Silva L, Damasceno H (2015). Health-related quality of life of students from a private medical school in Brazil. Int J Med Educ.

[10] Escola Bahiana de Medicina e Saúde Pública. Curso de Medicina. https://www.bahiana.edu.br/graduacao/cursos/2154/medicina. Accessed 12 July 2015.

[11] Bertolazi AN, Fagondes SC, Hoff LS, Pedro VD, Barreto SSM, Johns MW (2009). Portuguese-language version of the Epworth sleepiness scale: validation for use in Brazil. J Bras Pneumol.

[12] Ciconelli RM, Ferraz MB, Santos W, Meinão I, Quaresma MR (1999). Tradução para a língua portuguesa e validação do questionário genérico de avaliação de qualidade de vida SF-36 (Brasil SF-36). Rev Bras Reumatol.

[13] Saris-Baglama RN, Dewey CJ, Chisholm GB (2010). QualityMetric Health OutcomesTM Scoring Software 4.0.

[14] Zhang Y, Ou F, Gao S, Gao Q, Hu L, Liu Y (2013). Effect of low income on health-related quality of life: a cross-sectional study in Northeast China. Asia Pac J Public Health.

[15] Paro HBMS, Morales NMO, Silva CHM (2010). Health-related quality of life of medical students. Med Educ.

[16] Fallahzadeh H, Mirzaei H (2012). Health-related quality of life and associated factors among Iranian university students. J Community Health Res.

[17] OPTUM. SF Health Surveys. https://www.optum.com/optum-outcomes/what-we-do/health-surveys.html. Accessed 12 July 2015.

[18] Raj SR, Simpson CS, Hopman WM, Singer MA (2000). Health-related quality of life among final-year medical students. Can Med Assoc J.

[19] Jamali A, Tofangchiha S, Jamali R (2013). Medical students’ health-related quality of life: roles of social and behavioural factors. Med Educ.

[20] Pekmezovic T, Popovic A, Tepavcevic DK, Gazibara T, Paunic M (2011). Factors associated with health-related quality of life among Belgrade university students. Qual Life Res.

[21] Sales EC, Santana VS (2003). Depressive and anxiety symptoms among housemaids. Am J Ind Med.

[22] DIEESE – Departamento Intersindical de Estatística e Estudos Socioeconômicos (2013). O Emprego Doméstico no Brasil.

[23] Rodrigues RND, Viegas CAA, Abreu e Silva AAA, Tavares P (2002). Daytime sleepiness and academic performance in medical students. Arq Neuro-psiquiat.

[24] Ferri-de-Barros JE, Alencar MJ, Berchielli LF, Castelhano Júnior LC (2011). Headache among medical and psychology students. Arq Neuro-psiquiat.

